# A reassessment about included studies and certainty of evidence on a systematic review and meta-analysis of steroid for patients with acute respiratory distress syndrome

**DOI:** 10.1186/s40560-021-00529-9

**Published:** 2021-03-16

**Authors:** Shodai Yoshihiro, Shunsuke Taito

**Affiliations:** 1 Pharmaceutical department, JA General Hospital, 1-3-3 Jigozen, Hatsukaichi-shi, Hiroshima 738-8503 Japan; 2 Division of Rehabilitation, Department of Clinical Practice and Support, Hiroshima University Hospital, 1-2-3, Kasumi, Minami-ku, Hiroshima 734-8551 Japan

**Keywords:** Adult respiratory distress syndrome, Corticosteroids, Adrenal cortex hormone, Critical care, Respiratory failure

## Abstract

We comment on the study by Hirano et al. about the effect of steroids in patients with acute respiratory distress syndrome. It might be necessary to include only the existing randomized control trials and to reassess the certainty of evidence about the primary outcomes.

## Main text

Hirano et al. reported that prolonged administration of steroids to patients with early onset of acute respiratory distress syndrome (ARDS) was associated with improved mortality [1]. This study is a valuable report that suggests the need to extend the duration of steroid administration for patients with ARDS. However, it is possible that this study is not a systematic review based on the best available evidence. Therefore, we would like to focus on two concerns related to the primary outcomes of this systematic review and meta-analysis.

Only randomized control trials (RCTs) published on 1 August 2020 should be included in the systematic review. Annanne et al. performed a post hoc analysis of RCTs and did not stratify patient allocation by patients with ARDS in their original study [2]. The authors’ search method did not describe about statements to exclude articles that were published in languages other than English, although the RCT of Liu et al. was not included [3]. In addition, the RCT of Rezk et al. may need to be assessed for eligibility [4]. It might be necessary to re-synthesize studies to include only the existing RCTs.

We believe that the certainty of evidence about the primary outcomes may be much lower. The imprecision and risk of bias of 60- and 28- or 30-day mortality rates were evaluated as “not serious.” The 60-day mortality did not meet the optimal information size (OIS) because the total number of events was 179. In 28- or 30-day mortality, when the study by Annane et al. was excluded and Liu's study was included (4 RCTs, *N* = 591), the relative risk reduction was 35%, which did not appear to meet the OIS criteria. Downgrades might be needed. A pre-protocol was not published in the study of Meduri et al; therefore, the selective reporting bias was unclear. Although, the Egger’s test was used to analyze publication bias, it was not appropriate when the number of studies was less than 10 because of insufficient power [5]. An appropriate response would be to check the number of studies that have not been published on the Clinical Trials.gov and the International Clinical Trials Registry Platform.

We suggest reassessing the certainty of evidence regarding the primary outcome.

## Data Availability

Not applicable.

## Abbreviations

ARDS: Acute respiratory distress syndrome
RCT: Randomized control trial
